# Complete mitochondrial genome of *Dacus trimacula* (Diptera: Tephritidae) using next-generation sequencing from China

**DOI:** 10.1080/23802359.2019.1629351

**Published:** 2019-07-12

**Authors:** Yan-Ling Ren, Tao Wang, Dai Renhuai, Zhi-Yi Wu, Juan Wang

**Affiliations:** a The Research Center for Guizhou Characteristic Fruits and Its Products of Mountainous Regions, GuiZhou Institute of Light Industry, Guiyang, P. R. China;; b Institute of Entomology, Guizhou University, Guiyang, P. R. China;; c Zhejiang Academy of Science and Technology for Inspection and Quarantine, Hangzhou, P. R. China

**Keywords:** Mitogenome, Dacinae, *Dacus trimacula*, phylogeny

## Abstract

The complete mitochondrial genome (mitogenome) of the fruit fly species *Dacus trimacula* (Diptera: Tephritidae: Dacinae) are sequenced and annotated. The mitochondrial genome is 15,851 bp (GenBank No. MK940811) has an A + T content of 72.8% (A 39.2%; C 17.0%; G 10.2%, and T 33.6%), which is the classical structure for insect mitogenome. All PCGs started with ATN and stopped with TAN. The phylogenetic tree confirms that *D. trimacula* clustered with *D. longicornis* as the sister group to *Zeugodacus*. This study enriches the mitogenomes of the fruit flies.

The genus *Dacus* Fabricius belongs to the tribe Dacini of subfamily Dacinae (Diptera: Tephritidae). This genus includes more than 200 species which is one of the most economically important fruit flies; and, most *Dacus* species eat the pods of Asclepiadaceae and Apocynaceae or the fruits and flowers of Cucurbitaceae (Drew 2004). The majority of *Dacus* distribute in the African, and several species are found in the Indian Subcontinent, Southeast Asia, Australia, and Pacific (Drew 2004; Jiang et al. 2016). In the present study, we have sequenced and determined the complete mitochondrial genome (mitogenome) using next-generation sequencing method for the first time, which obtained for fruit flies in this study will facilitate future studies on the identification, population genetics, and evolution of the subfamily Dacinae.

Total genome DNA was extracted from male adult of *Dacus trimacula* which was collected in Qingyan ancient town, Guizhou province, China (106°7′E, 26°3′N), in July 2018. And, voucher specimen’s genome DNA is deposited in the Institute of Entomology, Guizhou University, Guiyang, China (GUGC). The complete mitogenome of *D. trimacula* is 15,851 bp (GenBank No. MK940811), containing 13 protein-coding genes (PCGs, 11,193 bp), 22 transfer RNA genes (tRNAs, 1469 bp), two ribosomal RNA genes (rRNAs, 2121 bp), and a large non-coding region (Control region, 944 bp). In summary, the *D. trimacula* mitogenome has an A + T content of 72.8% (A 39.2%; C 17.0%; G 10.2% and T 33.6%). All PCGs started with ATN and terminated with TAN; all tRNAs are identified using ARWEN version 1.2 (Laslett and Canbäck 2008); the 16S rRNA gene is located between *tRNA-L2* and *tRNA-V* with 1327 bp, and *12S* rRNA gene is 794 bp in length and is located between *tRNA-V* and Control region.

The phylogenetic relationships of *D. trimacula* were reconstructed with IQ-TREE using an ultrafast bootstrap approximation approach with 10,000 replicates based on concatenated the first and second codon positions of the 13 PCGs and two rRNAs with 9494 nucleotides (Figure 1). Each PCG and rRNA sequence was aligned using the MAFFT algorithm in TranslatorX and MAFFT v7.0 online serve with the G-INS-i strategy respectively, and aligned sequences were eliminated using Gblocks 9.1b (Abascal et al. 2010; Katoh et al. 2017). The phylogenetic tree confirms that *D. trimacula* clustered with *D. longicornis* as the sister group to *Zeugodacus*. Up to now, few studies have been recorded for Deltocephalinae, and we hope that our data can be useful for further study.

**Figure 1.**
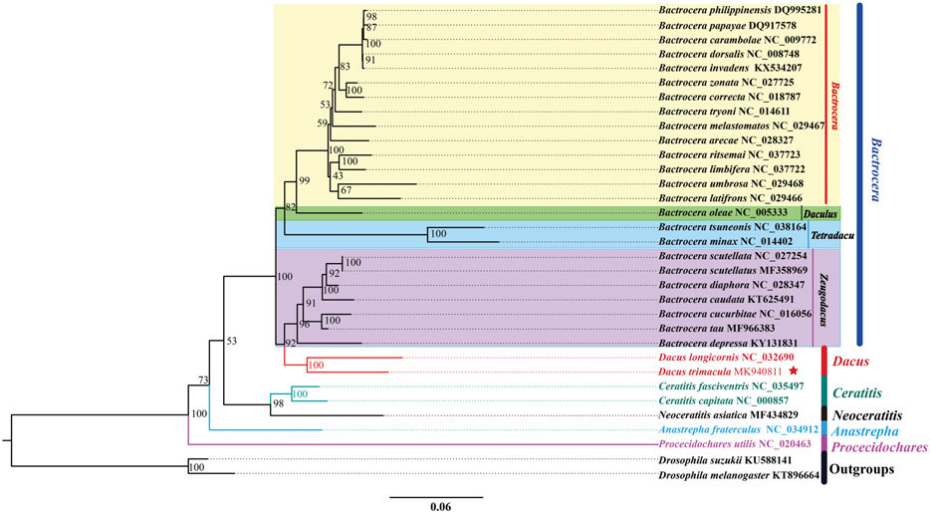
Phylogenetic analyses of *Dacus trimacula* based upon the concatenated the first and second codon positions of the 13 PCGs and two rRNAs of 31 ingroup species. The analysis was performed using IQ-TREE software. Numbers at nodes are bootstrap values. The accession number for each species is indicated after the scientific name.
